# Safety Evaluation and Anti-Inflammatory Efficacy of *Lacticaseibacillus paracasei* PS23

**DOI:** 10.3390/ijms24010724

**Published:** 2022-12-31

**Authors:** Chin-Hao Li, Tai-Ying Chen, Chien-Chen Wu, Shih-Hsuan Cheng, Min-Yu Chang, Wei-Hong Cheng, Shih-Hau Chiu, Chien-Chi Chen, Ying-Chieh Tsai, Deng-Jye Yang, Jaw-Jou Kang, Po-Lin Liao

**Affiliations:** 1 Graduate Institute of Medical Sciences, College of Medicine, Taipei Medical University, Taipei 110, Taiwan; 2 Department of Physiology, School of Medicine, College of Medicine, Taipei Medical University, Taipei 110, Taiwan; 3 Graduate Institute of Toxicology, College of Medicine, National Taiwan University, Taipei 100, Taiwan; 4 Bened Biomedical Co., Ltd., Taipei 104, Taiwan; 5 Institute of Biochemistry and Molecular Biology, National Yang Ming Chiao Tung University, Taipei 112, Taiwan; 6 Bioresource Collection and Research Center, Food Industry Research and Development Institute, Hsinchu 300, Taiwan; 7 Institute of Food Safety and Health Risk Assessment, School of Pharmaceutical Sciences, National Yang Ming Chiao Tung University, Taipei 112, Taiwan; 8 Toxicological Research Laboratory, School of Pharmaceutical Sciences, National Yang Ming Chiao Tung University, Taipei 112, Taiwan

**Keywords:** *Lacticaseibacillus paracasei* PS23, genome-based safety assessment, 28-day repeated oral dose toxicity, genotoxicity test, no observed adverse effect level

## Abstract

*Lacticaseibacillus paracasei* strain PS23 (PS23) exhibits some probiotic properties. In this study, a genomic analysis of PS23 revealed no genes related to virulence or antibiotic resistance. Moreover, ornithine decarboxylase activity was not detected in vitro. In addition, PS23 was sensitive to the tested antibiotics. Genotoxicity tests for PS23 including the Ames test and chromosomal aberrations in vitro using Chinese hamster ovary cells and micronuclei in immature erythrocytes of ICR mice were all negative. Moreover, following a 28-day study involving repeated oral dose toxicity tests (40, 400, and 4000 mg/kg equal 1.28 × 10^10^, 1.28 × 10^11^, and 1.28 × 10^12^ CFU/kg body weight, respectively) using an ICR mouse model, no adverse effects were observed from any doses. In addition, supplementation with live or heat-killed PS23 ameliorates DSS-induced colonic inflammation in mice. Our findings suggest that PS23 is safe and has anti-inflammatory effects and may therefore have therapeutic implications.

## 1. Introduction

Probiotics are live microorganisms that, when administered in adequate amounts, confer a health benefit on the host [1]. Probiotics contain both live yeast and bacteria (including *Lactobacillus, Pediococcus, Bifidobacterium,* and *Bacillus*) genera [2]. Several studies have shown that probiotics have diverse beneficial effects [3] such as the moderation of various disorders, including the regulation of immune responses [4], anti-tumor effects [5], cardiovascular protection [6,7], attenuation of diabetes [8], relief of depression [9], anti-obesity effects [10], and alleviation of diarrhea [11]. Probiotics may also exert modulatory effects against the COVID-19 virus [12]. 

Various strains of *Lacticaseibacillus paracasei* have beneficial effects, including the prevention of inflammatory responses and hyperlipidemia, as well as lowering cholesterol [13,14,15]. Recently, an *L. paracasei* strain, PS23, isolated from healthy human feces, has demonstrated stress-relieving and anti-aging potential [16,17,18,19]. Evidence has demonstrated that administering PS23 improves behavioral responses against psychological characteristics using a maternal separation model [18]. A senescence-accelerated mouse model demonstrated that long-term administration of PS23 may delay the progression of anxiety-like behaviors and memory impairment during aging [17]. Moreover, PS23 may inhibit sarcopenia progression in aging by decreasing age-related inflammation and reactive oxygen species, followed by maintaining mitochondrial function [16]. A previous study also indicated that PS23 could become a potential psychobiotic for major depressive disorder management by reversing chronic corticosterone-induced anxiety and depression-like behaviors [19].

Inflammatory bowel disease (IBD) refers mainly to Crohn’s disease; however, and ulcerative colitis (UC) is one of the main causes of IBD-associated deaths, with approximately 38,000 deaths reported in 2017 [20]. Patients with IBD are at an elevated risk of developing colorectal cancer, and the sustained increase in its prevalence increases the global economic burden [21]. A high proportion of patients respond poorly to the current treatment options for IBD; therefore, potential alternative therapeutic agents and/or food supplements targeting IBD are needed [22]. In recent years, probiotics have been considered potentially beneficial in patients with IBD owing to their abilities to restore intestinal microbial balance, improve gut barrier function, and decrease inflammatory responses [23]. However, whether PS23 can alleviate colitis in vivo remains unknown.

Although probiotics are extensively available to consumers and are commonly purchased as food probiotics (yogurts, cheeses, milk-based beverages, fermented fish, meat, and vegetables) or food supplements (tablets, capsules, pills, powders, liquid concentrates in vials, and soft gels), the safety of their usage remains unclear, and there are few reports on the side effects of probiotics, including those of lactobacilli [24,25]. Therefore, in this study, we evaluate the genome-based safety, antibiotic resistance, genotoxicity, and 28-day subacute toxicity of *L. paracasei* strain PS23, as well as its possible therapeutic potential against IBD.

## 2. Results and Discussion

### 2.1. Genome-Based Safety Assessment of L. paracasei PS23

Sequencing statistics, including total raw bases and the number of reads for strain PS23 obtained via PacBio, are summarized in Table S1. The assembled genome consisted of two contigs (one chromosome and one plasmid-like contig) with a genome size of 3.01 Mb and a GC content of 46.3%. Gene prediction and annotation results indicated that the genome contains 2890 protein coding genes, 15 rRNAs, and 59 tRNAs (Table S2). The whole-genome sequence of strain PS23 was compared against up-to-date antibiotic resistance genes and virulence factor databases, and no suspected genes with a high similarity were found (Tables S3 and S4). In addition, the whole genome sequence of strain PS23 was compared against the BLAST database for any existing biogenic amine-producing genes, including histidine decarboxylase, tyrosine decarboxylase, ornithine decarboxylase, agmatine deiminase, and lysine decarboxylase. The results showed only one gene homologous to a known ornithine decarboxylase [EC:4.1.1.17], which may participate in the biosynthesis of putrescine (Table S5). 

### 2.2. L. paracasei PS23 Did Not Exhibit Ornithine Decarboxylase Activity In Vitro

Some lactobacilli have reportedly produced biogenic amines [26]. Oral exposure to biogenic amines at higher concentrations may cause adverse effects among the general public [27]. The results from the genome-based safety assessment of *L. paracasei* PS23 indicated that ornithine decarboxylase can be produced (Table S5). Moreover, the sequence of this enzyme is widespread across many *L. paracasei* strain genomes deposited in public domain databases. To address safety issues, the ability of *L. paracasei* PS23 to decarboxylate ornithine and produce putrescine was experimentally confirmed by an in vitro assay. As shown in Figure S1, the Moeller decarboxylase broths inoculated with 1% (*v*/*v*) fresh MRS broth or overnight grown lactobacilli culture were light brown and dark yellow in color, respectively. After 96 h of anaerobic incubation at 37 °C, media inoculated with lactobacilli turned purple (decarboxylase positive) or bright yellow (decarboxylase negative) in color. Ornithine decarboxylase activity was detected in *L. saerimneri* 30a and *L. paracasei* ATCC 25302^T^ but not in *L. casei* ATCC 393^T^ and *L. paracasei* PS23. These results demonstrate that, under the assay condition, PS23 did not exhibit ornithine decarboxylase activity.

### 2.3. Antibiotic Resistance Profile of L. paracasei PS23

The genomic analysis did not find any antibiotic resistance genes in PS23 (Table S3). MICs for the antibiotics compared against the PS23 treatment and the EFSA cutoff values of *L. paracasei* species are shown in Table 1. PS23 exhibited MIC values lower than the MIC breakpoint values recommended by EFSA for the antibiotics tested (ampicillin, gentamicin, kanamycin, streptomycin, erythromycin, clindamycin, tetracycline, and chloramphenicol). Since PS23 was not resistant to the tested antibiotics, no further studies on their antibiotic resistance are required according to the EFSA.

### 2.4. L. paracasei PS23 Evaluation for Mutagenicity and Clastogenicity In Vitro and In Vivo

The Ames test revealed that the number of revertants did not increase after treatment with PS23 (0.3125, 0.625, 1.25, 2.5, and 5 mg/plate), with or without S9 metabolic activation, in all five test strains (TA97, TA98, TA100, TA102, and TA1535). Conversely, a significantly higher number of revertants was observed in the positive control group (Table 2). In the MTT assay, CHO-K1 cells were treated with PS23 (0, 0.078, 0.16, 0.313, 0.625 and 1.25 mg/mL) for 24 h, and the cell viability values were 100 ± 0.0, 95.3 ± 5.9, 88.5 ± 4.8, 80.8 ± 4.0, 58.5 ± 5.4, and 18.2 ± 3.8, respectively. PS23, at a dose of 0.625 mg/mL, retained cell viability at 58.5 ± 5.4 following treatment for 24 h; therefore, it was set as the highest treatment group for the chromosomal aberration test. The results indicated that none of the PS23 treatment groups showed any abnormal increase in the number of abnormal chromosomes (Table 3). The ratio of polychromatic erythrocytes (PECs) to total erythrocytes (%) and the frequency of micronucleated polychromatic erythrocytes (MNPECs) (‰) in the treatment groups (0.5, 1.0, and 2.0 g/kg body weight) obtained via the micronucleus assay showed no significant increase compared to the negative control group treated with the same amount of vehicle (distilled water, Table 4). These data show that PS23 did not cause mutagenicity or clastogenicity.

### 2.5. L. paracasei PS23 Evaluation for Adverse Effects in the Subacute Toxicity Study

Following 28 days of repeated oral administration of PS23 powder, the body weight of each group increased normally, and no significant difference was found between any of the groups (0, 40, 400, and 4000 mg/kg body weight). No mortality or clinical signs of toxicity were observed. The weekly food and water consumption rates in the 40 mg/kg, 400 mg/kg, and 4000 mg/kg groups showed no significant difference compared with the control group. The results of the blood component analysis are presented in Table 5. Significant differences (*p* < 0.05) were observed in platelet distribution width (PDW) and mean platelet volume (MPV) in the male groups at 40 mg/kg dosage, whereas the platelet large cell ratio (P-LCR) from female groups at 40 mg/kg and 400 mg/g treatment showed significant differences compared to the control groups; however, there was no dose-dependent difference. The results of the biochemical analyses of the blood samples are shown in Table 6. In the male mice, the values of sodium, cholesterol, and amylase in the 40 mg/kg PS23 treatment group, the phosphorous and aspartate aminotransferase (AST) values in the 4000 mg/kg PS23 treatment group, the glucose value in the 40 mg/kg and 400 mg/kg PS23 treatment groups, and the chloride values in all treatment groups were significantly different from those of the control group (*p* < 0.05). In the female mice, the sodium values for the 40 mg/kg PS23 treatment group and the chloride and alkaline phosphatase (ALP) values were significantly different from those of the control group (*p* < 0.05). None of these changes from the biochemical analyses were dose-dependent; hence, they were not considered as toxic responses.

### 2.6. Lack of Histopathological Defects Following Subacute Toxicity Study for L. paracasei PS23

**Figure 1:**
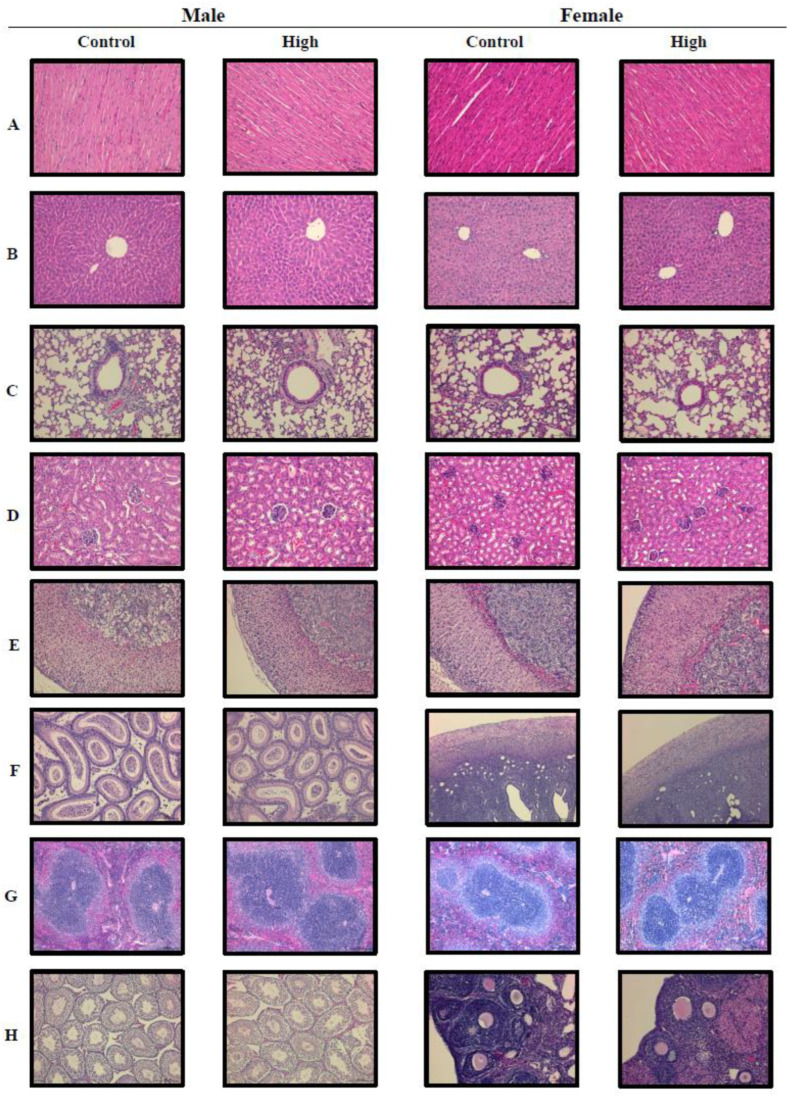
Analysis of histopathology in the ICR mice from the *L. paracasei* PS23 28-day toxicity test. Representative images of the histopathological demonstration for the male control ICR mice and the high dose (4000 mg/kg) groups include the heart (**A**), liver (**B**), lung (**C**), kidneys (**D**), adrenal glands (**E**), epididymis (**F**), spleen (**G**), and testis (**H**) (H&E stain, 200×) (left panel). Representative pictures from the female control ICR mice and the high-dose (4000 mg/kg) groups include the heart (**A**), liver (**B**), lung (**C**), kidneys (**D**), adrenal glands (**E**), uterus (**F**), spleen (**G**), and ovaries (**H**) (H&E stain, 200×) (right panel).

After the mice were euthanized, the heart, lung, liver, spleen, kidneys, adrenal glands, and gonads (male: epididymis and testis; female: ovary and uterus) were carefully collected and weighted. The results are listed in Table 7. Histological assessment of the heart, lung, liver, spleen, kidneys, adrenal glands, and gonads (male: epididymis and testis; female: ovary and uterus) was performed to estimate the effect of 28-day repeated oral administration of *L. paracasei* PS23. Although treatment of 400 mg/kg PS23 powder in the female groups and 40 mg/kg and 400 mg/kg PS23 powder in the male groups resulted in significantly different results to the control group for the liver organ weight and the ratio to body weight (Table 7), the changes indicated no dose-dependency. More importantly, no histopathological abnormality was observed in all three treatment groups in both male and female mice when compared with the control group. Representative histopathological pictures of the control and the high-dose (4000 mg/kg) group are shown in Figure 1. Therefore, a no-observed-adverse-effect level (NOAEL) was established in mice at a 4000 mg/kg body weight. As the viability of PS23 powder was approximately 3.2 × 10^11^ CFU/g, the supplemented dose equated to 2.6 × 10^10^ CFU/20 g mouse/day, which corresponds to a human equivalent amount of 6.2 × 10^12^ CFU/60 kg person/day according to an appropriate animal-specific body surface area-based conversion [29].

### 2.7. L. paracasei PS23 Ameliorates Colonic Inflammation Induced by DSS

**Figure 2:**
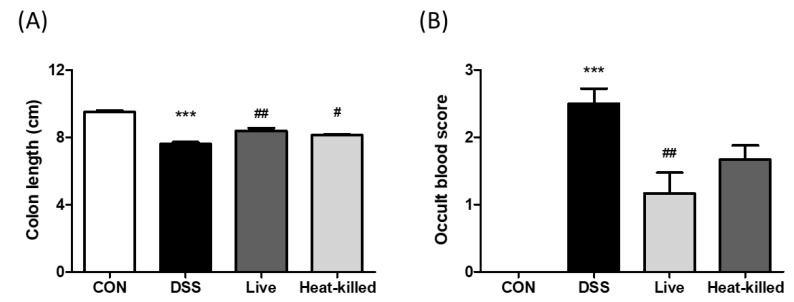
Oral administration of *L. paracasei* PS23 ameliorates shortened colon length and intestinal bleeding in DSS-induced colitis mice. At end of the experiment, the colon tissues were removed to measure the colon length (**A**). Fecal occult blood scores were also tested (**B**). Data are presented as mean ± SD. *** *p* < 0.001 versus the CON group, and # *p* < 0.05, and ## *p* < 0.01 versus the DSS group.

**Figure 3:**
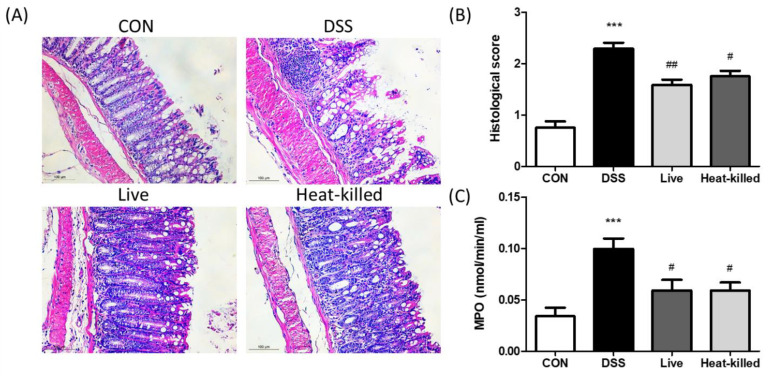
Hematoxylin–eosin-stained colon sections (**A**), histological score (**B**), and myeloperoxidase (MPO) activity of colon tissues (**C**) in DSS-induced colitis mice. Data are presented as mean ± SD. *** *p* < 0.001 versus the CON group, and # *p* < 0.05 and ## *p* < 0.01 versus the DSS group.

**Figure 4:**
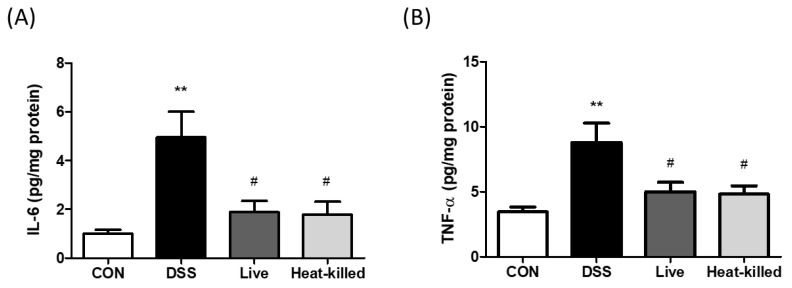
Levels of pro-inflammatory cytokines IL-6 (**A**) and TNF-α (**B**) in the colon tissue of DSS-induced colitis mice. Data are presented as mean ± SD. ** *p* < 0.01 versus the CON group, and # *p* < 0.05 versus the DSS group.

DSS-treated animals developed UC in a previous study and were characterized by colon shortening, intestinal bleeding, crypt damage, and leukocyte infiltration into the mucosa [30,31]. In this study, in comparison, we used a DSS-induced UC mouse model to investigate the anti-inflammatory effects of *L. paracasei* PS23 (*3.14. Dextran Sulfate Sodium (DSS)-Induced Colitis in Mice*). Compared with the CON group, the DSS treatment significantly shortened colon lengths (Figure 2A) and increased rectal bleeding scores in the occult blood test (Figure 2B), whereas these UC symptoms were ameliorated through both live and heat-killed PS23 supplementation. These results are in line with a previous finding that milk fermented with *Lactobacillus delbrueckii* subsp. *bulgaricus*, *Streptococcus thermophilius*, and *L. paracasei* PS23 exhibited anti-colitis effects [32]. Colon length shortening in DSS-treated mice is a biological marker in assessments of colonic inflammation [33], and disrupting the intestinal barrier function may lead to occult hemoglobin in the feces [34]. Thus, PS23 may improve gut permeability and reduce gut inflammation through exerting anti-colitis effects. Furthermore, DSS treatment significantly increased colon tissue damage and leukocyte infiltration, as assessed by histological observations (Figure 3A), scoring (Figure 3B), and colonic MPO activity (Figure 3C). These effects were ameliorated by supplementation with both live and heat-killed PS23. MPO is derived from neutrophils and associated with inflammatory disorders [35]. A previous study suggested that PS23 protects colonic tissues from damage by inhibiting neutrophil recruitment and infiltration [36]. In addition, the colonic levels of pro-inflammatory cytokines, IL-6 and TNF-α, were evaluated, as they may act as important mediators in IBD [37] and may promote inflammatory responses by stimulating neutrophil migration and promoting intestinal epithelial tissue damage [38]. Compared to the CON group, the levels of IL-6 (Figure 4A) and TNF-α (Figure 4B) were significantly increased in the DSS group in this study, and this result was reversed in both live and heat-killed PS23 supplementation. These results suggest that PS23 could be used to prevent and treat chronic diseases associated with intestinal inflammation.

## 3. Materials and Methods

### 3.1. Preparation of L. paracasei PS23

*Lacticaseibacillus paracasei* subsp. *paracasei* (previously classified as *Lactobacillus paracasei* subsp. *paracasei*) PS23 was isolated from the feces of healthy humans [18] and routinely cultivated in a De Man, Rogosa, and Sharpe (MRS) medium (Difco Laboratories, Sparks, MD, USA) at 37 °C. The lyophilized PS23 powder, containing approximately 3.2 × 10^11^ colony-forming units (CFU)/g of PS23, was provided by Bened Biomedical Co., Ltd. and stored at −20 °C. The PS23 powder was resuspended and adjusted to the desired concentration in phosphate-buffered saline (PBS) before use.

### 3.2. Genome-Based Safety Assessment of L. paracasei PS23

Details of genome sequencing and assembly, identifications of virulence- and antibiotic-resistance-related genes, and detections of biogenic amine-producing genes are provided in the Supplementary File [39,40,41,42,43,44,45,46,47,48,49,50,51]. The complete genome sequence of *L. paracasei* PS23 (3,014,784 bp) was made available in the GenBank database under the nucleotide sequence accession numbers CP078097 (chromosome, 2,939,246 bp) and CP078098 (plasmid-like contig., 75,538 bp).

### 3.3. Assessment of Ornithine Decarboxylase Activity of Lactobacilli

The ornithine decarboxylase activity of lactobacilli was evaluated as previously described, with minor modifications [52]. In brief, lactobacilli were sub-cultured in MRS broth, and the overnight grown cultures were 1% (*v*/*v*) inoculated into wells containing Moeller decarboxylase broth (HiMedia Laboratories, India) supplemented with 2% L-ornithine monohydrochloride (Sigma-Aldrich, St. Louis, MO, USA) in a 96-well plate. The final pH of the uninoculated Moeller decarboxylase broth was adjusted to 6.0 ± 0.2. The wells were overlaid with sterile mineral oil, and then the plate was incubated anaerobically at 37 °C for 96 h. In general, the formation of putrescine, a biogenic amine, from ornithine increases the pH of the medium, changing the color of the indicators (bromo cresol purple and cresol red) to purple. If lactobacilli fermented glucose and form acidic byproducts but did not produce ornithine decarboxylase, the medium would then be acidic and yellow. The appearance of a yellow broth is an indication of glucose fermentation, not decarboxylation. A Moeller decarboxylase broth supplemented with 1% fresh MRS broth was used as the blank control in this study. *Lactobacillus saerimneri* 30a (ATCC 33222) with ornithine decarboxylase activity was used as the positive control [53]. *L. paracasei* PS23 and two taxonomic-type strains, *Lacticaseibacillus casei* ATCC 393^T^ and *L. paracasei* ATCC 25302^T^, were evaluated.

### 3.4. Antibiotic Resistance Profile of L. paracasei PS23

The antibiotic resistance profile of PS23 was evaluated according to the standard method procedure ISO 10932 [54]. The minimum inhibitory concentration (MIC) was determined after 48 h of growth at 28 °C in an anaerobic chamber (Coy Laboratory Products, Grass Lake, MI, USA, atmosphere 85% N_2_, 5% CO_2_, 10% H_2_) for the following antimicrobial agents, as recommended by the EFSA [28]. *L. paracasei* ATCC 25302^T^ was used as the reference strain for quality control [54]. 

### 3.5. Bacterial Reverse Mutation Test

*Salmonella* Typhimurium histidine strains TA 98, TA 100, TA 102, TA 1537, and TA 1535 were obtained from Moltox (TN, USA). Mutagenicity using *Salmonella* Typhimurium was assessed in the presence or absence of rat liver post-mitochondrial (S9) metabolic activation [55]. Briefly, a test tube was mixed with PS23, 100 mL of each test bacterial culture (10^9^ cells/mL), 2 mL soft agar (0.6% agar, 0.5% NaCl, 5 mM histidine, and 50 mM biotin, pH 7.4, 40–50 °C), and 0.5 mL S9 mixture (if necessary). Following mixing, the samples were placed on minimal agar plates (1.5% agar, Vogel-Bonner E medium containing 2% glucose). The plates were incubated for 48 h at 37 °C in the dark. The revertant colonies were counted macroscopically. 

### 3.6. Cytotoxicity Assay

The 3-(4,5-dimethylthiazol-2-yl)-2,5-diphenyltetrazolium bromide (MTT) assay was used to measure cellular metabolic activity as an indicator of cell viability in Chinese hamster ovary (CHO) cells. CHO cells were cultured in 96-well plates and treated with PS23 as described previously [56]. After 24 h, MTT solution (0.5 mg/mL) was added to each well for 1 h. Dimethyl sulfoxide (DMSO) was used to dissolve the formazan crystals, and the absorbance of each well was read at 590 nm. Cell viability was calculated relative to that of the untreated control group.

### 3.7. Analysis of Chromosomal Aberrations 

To analyze chromosomal abnormalities, CHO epithelial cells (CHO-K1, cat. no. CCL-61, American Type Culture Collection, Manassas, VA, USA) were cultured in 6 cm dishes at a density of 2 × 10^5^ cells/mL. The cells were treated with distilled water, mitomycin C (1 μg/mL), benzo(a)pyrene (5 μg/mL), or PS23 (0.625, 1.25, or 2.5 mg/mL), with or without S9. Next, 0.1 μg/mL Colcemid was administered for 3 h before trypsinization, followed by cell collection and treatment with 0.9% sodium citrate at 37 °C and Carnoy’s solution (methanol: acetic acid, 3:1). The cells were spread on glass slides and stained with 3% Giemsa solution in a 0.07 M phosphate buffer (pH 6.8). On each slide, 100 well-spread cells were counted for chromosomal alterations. Data are expressed as the mean number of chromosomal aberrations per treatment ± standard deviation (SD) from 300 cells scored in three independent experiments.

### 3.8. Ethics Statement 

Male ICR mice (8 weeks old upon arrival, N = 25, National Laboratory Animal Center, Taipei, Taiwan) were randomly assigned to five cages for an in vivo mammalian erythrocyte micronucleus test; other ICR mice (8 weeks old upon arrival, 40 male and 40 female, National Laboratory Animal Center, Taipei, Taiwan) were randomly assigned to cages with five animals/cage and used to conduct a subacute (28 days) oral toxicity assessment. BALB/c mice (8 weeks old upon arrival, N = 24 National Laboratory Animal Center, Taipei, Taiwan) were randomly assigned to four cages for the efficacy evaluation in a dextran sulfate sodium (DSS)-induced colitis model. The animals were kept under a 12 h light/dark cycle at 22 ± 2 °C and 39–43% relative humidity and had ad libitum access to water and food (Rodent LabDiet 5001; PMI Nutrition International, LLC, Richmond, IN, USA). All of the experimental procedures involving the use of animals complied with the “Guide for the Care and Use of Laboratory Animals” (National Academy of Sciences Press, 1996). The experimental design was approved by the Institutional Animal Care and Use Committee (IACUC) of the National Yang Ming Chiao Tung University (approval numbers: 1041112, 1060606, and 1080333). All of the surgeries were performed under isoflurane anesthesia, and all possible efforts were made to minimize suffering. The animal testing criteria mandated that a loss of >20% of body weight compared with the pre-study weight was considered a humane endpoint in this study; none of the tested mice reached this endpoint during our study.

### 3.9. Mammalian Erythrocyte Micronucleus Test 

Male ICR mice (n = 5) were treated with PS23 via oral gavage at doses of 0.5, 1, and 2 g/kg body weight. The positive controls were administered with cyclophosphamide (200 mg/kg body weight) intraperitoneally. Blood samples (10 μL) were collected from the tail vein at 24, 48, and 72 h after dosing onto a slide pre-stained with acridine orange (40 μg/mL) and examined by fluorescence microscopy with a blue excitation wavelength and yellow-to-orange barrier filter (e.g., 515 nm long pass). The ratio (%) of polychromatic erythrocytes (PCEs) to total erythrocytes and the frequency of micronucleated polychromatic erythrocytes (MNPCEs) were calculated by counting a total of 1000 erythrocytes or PCEs per animal.

### 3.10. Subacute Toxicity Study 

Forty adult ICR mice (8 weeks old) were randomly separated into four groups and treated with PS23 (0, 1.28 × 10^10^, 1.28 × 10^11^, and 1.28 × 10^12^ CFU/kg body weight, corresponding approximately to 0, 1, 10, and 100 times the recommended daily intake in humans) via gavage for 28 days. The recommended daily intake of PS23 in commercially available health food and clinical research (https://clinicaltrials.gov/, identifier: NCT04971096) is 6 × 10^10^ CFU. The highest dose used in the study was 4000 mg/kg body weight which corresponds to 6.2 × 10^12^ CFU (100 times higher than the recommended daily intake of PS23) for a 60 kg person according to an animal specific body surface area-based conversion [27]. Mortality and abnormal signs were observed twice daily. Body weights were measured twice per week. Food and water consumption rates were measured at 2 and 4 weeks. On day 29, all of the mice were sacrificed. Cage-side observations were conducted daily and included eye movements, respiration, fur, motion, and the activity rates of all animals. 

### 3.11. Hematological and Serum Biochemical Analyses 

At the end of the 28-day toxicity test, the ICR mice fasted overnight and were placed under general anesthesia with isoflurane (2% with a Matrx VIP-3000 veterinary anesthetic vaporizer). Blood samples were collected from the abdominal aorta for hematology, coagulation, and serum chemistry tests; then, the samples were examined using an automatic analyzer. Sysmex XT-1800iv (TOA Medical Electronics Co., Kobe, Japan) was used for the comprehensive blood (CBC/DC) analysis. Clinical biochemical analysis was performed using Fuji DRI-CHEM slides GPT/ALT-PIII using a Fuji Dri-chem 4000i (FUJIFILM Corporation, Tokyo, Japan). 

### 3.12. Gross Necropsy

Gross necropsy was performed to analyze macroscopic external and internal features [57,58]. Vital organs were carefully removed, defatted, weighed individually, and fixed in 10% neutral-buffered formalin. The organ wet weights are expressed in absolute and relative terms. The fixed tissue segments were embedded in paraffin and stained with hematoxylin and eosin (H&E, Sigma, St. Louis, MO, USA) for histological assessment under a light microscope (ZEISS Axio Vert.A1, Leica Microsystems, Heerbrugg, Switzerland). After the mice were sacrificed, the heart, lung, liver, spleen, kidneys, adrenal glands, and gonads were carefully collected, and the organ weights were measured. The ratio of organ weights was calculated as follows: ratio of organ weight (%) = organ weight (g)/body weight (g) × 100.

### 3.13. Histopathology

Biopsy sections of the heart, lung, liver, spleen, kidneys, adrenal glands, and gonads were fixed with 4% formaldehyde in phosphate-buffered saline (PBS) at pH 7.4. The fixed tissue segments were embedded in paraffin and stained with H&E (Sigma) for histological assessment under a light microscope (Leica DM750). 

### 3.14. Dextran Sulfate Sodium (DSS)-Induced Colitis in Mice

**Figure 5:**
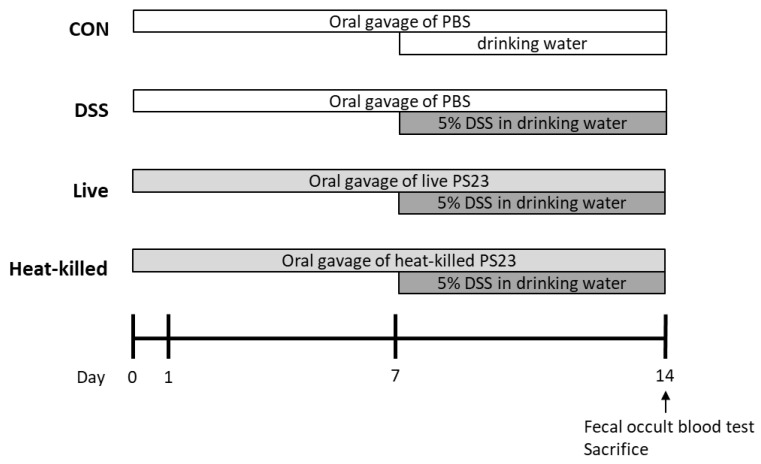
Experimental design of DSS-induced ulcerative colitis mouse model. Six-week-old female BALB/c mice were randomly assigned to four groups (n = six per group), including healthy control mice (CON), DSS-induced colitis mice (DSS), DSS-induced colitis mice supplemented with live PS23 (Live), and DSS-induced colitis mice supplemented with heat-killed PS23 (heat-killed). From days 1 to 14, the mice were orally administered PBS or *L. paracasei* PS23 (live or heat-killed form) via gavage, once per day. From days 8 to 14, the colitis mice were given 5% DSS in their drinking water and the control mice were given water only. On day 14, feces were collected for an occult blood test, and all of the mice were sacrificed for further evaluations.

The DSS-induced colitis mouse model was established as previously described [59]. Mice were adapted to the environment for at least 1 week prior to experimentation. The experimental design is illustrated in Figure 5. Twenty-four (6-week-old) female BALB/c mice were randomly divided into four groups (n = six per group): healthy control mice (CON), DSS-induced colitis mice (DSS), DSS-induced colitis mice supplemented with live PS23 (live), and DSS-induced colitis mice supplemented with heat-killed PS23 (heat-killed). During the experiment (days 1 to 14), the mice in the CON and DSS groups were given 0.2 mL PBS daily via oral gavage, whereas those in the PS23 groups were administered 0.2 mL live or heat-killed PS23 suspensions via oral gavage. The PS23 suspension was adjusted to 10^10^ CFU/mouse/day in PBS (10^9^ CFU/mouse/day). For the heat-kill treatment, the suspension was heat-treated at 100 °C for 20 min in a water bath and stored at −20 °C until further use. Mice in the DSS, live, and heat-killed groups were administered 5% (*w/v*) DSS (MW 36,000–50,000 Da, MP Biomedicals, Solon, OH, USA) dissolved in drinking water from days 8 to 14. Healthy control mice received water only from days 8 to 14. During feces collection, the mice were removed from their cages and placed individually in circular plastic containers with filter paper at the bottom. Approximately 150–200 mg fecal pellets per mouse were collected, and occult blood in the feces was measured using a Hemoccult Sensa (Beckman Coulter, Brea, CA, USA). On day 14, all of the mice were sacrificed, and their colons were removed and measured for length. A portion of the colonic tissue was fixed in 10% formaldehyde solution and the rest was stored at -80°C until analysis. To perform histological evaluation and scoring, fixed colon tissue samples were dehydrated in ethanol, embedded in paraffin wax, and sectioned (5 µm thickness). These sections were routinely stained with an autostainer (Leica Autostainer XL ST5010 staining system, Leica Biosystems, Wetzlar, Germany) and H&E. In the histological scoring, four fields of view were photographed for each sample. Each field was evaluated for tissue damage and inflammatory cell infiltration by two independent, well-trained histologists in a blinded manner. The histological scores were assessed as described by Kwak et al. based on the degree of maintenance of epithelial architecture, crypt damage, and infiltration of inflammatory cells around the colon tissue, as follows: [normal (0), mild (1), moderate (2), and severe (3)] [60]. 

### 3.15. Colonic Myeloperoxidase (MPO) Activity and Cytokine Production

Colonic MPO activity was measured from 100 μg of colonic sample protein using an MPO peroxide assay kit (Cayman Chemical, Ann Arbor, MI, USA). The production rate of colonic inflammatory cytokines was measured using the DuoSet ELISA Development System (R&D Systems, Minneapolis, MN, USA) according to the manufacturer’s instructions.

### 3.16. Statistical Analysis

Data are expressed as mean ± SD. The differences between the means were tested for statistical significance using one-way analysis of variance (ANOVA) followed by Tukey’s post-hoc test. A *p*-value of <0.05 was considered statistically significant.

## 4. Conclusions

In this study, genomic and in vitro analyses showed that *L. paracasei* PS23 showed no safety concerns regarding virulence genes, antibiotic resistance, or biogenic amine production. The results of the Ames test, chromosomal aberration assay, and mammalian micronucleus test showed that probiotic PS23 powder did not exert a genotoxic effect. In the 28-day subacute toxicity study, the NOAEL was >4000 mg/kg body weight for both male and female mice, which corresponds to a human equivalent amount of 6.2 × 10^12^ CFU/60 kg person/day. Furthermore, both live and heat-killed PS23 ameliorated DSS-induced colonic inflammation in mice. These results illustrate the safety and anti-inflammatory effects of PS23 in humans, thereby suggesting therapeutic potential.

## Figures and Tables

**Table 1:** Minimum inhibitory concentration (MIC) values of antibiotics to *L. paracasei* PS23.

Antibiotics	Cut-Off Values of *L. paracasei* ^a^ (mg/L)	PS23	
		MICs (mg/L)	Interpretation
Ampicillin	4	1	S
Gentamicin	32	4	S
Kanamycin	64	64	S
Streptomycin	64	16	S
Erythromycin	1	0.125	S
Clindamycin	4	0.25	S
Tetracycline	4	1	S
Chloramphenicol	4	4	S

^a^, according to the guidance recommended by the EFSA [28]. S, sensitive.

**Table 2:** Mutagenicity of PS23 powder in *Salmonella* Typhimurium TA97, TA98, TA100, TA102, and TA1535.

	TA97	TA98	TA100	TA102	TA1535
	Without S9 metabolic activation				
Negative ^1^	18 ± 4	29 ± 5	53 ± 7	25 ± 2	17 ± 3
Positive ^2^	315 ± 31 *	591 ± 65 *	1290 ± 12 *	422 ± 14 *	1327 ± 64 *
PS23 (mg/plate)					
5	31 ± 12	18 ± 4	68 ± 13	21 ± 3	16 ± 3
2.5	18 ± 0	15 ± 2	66 ± 4	27 ± 4	14 ± 4
1.25	22 ± 11	17 ± 1	65 ± 7	24 ± 5	14 ± 5
0.625	23 ± 3	18 ± 5	71 ± 1	23 ± 2	11 ± 1
0.3125	16 ± 10	23 ± 4	51 ± 5	24 ± 5	24 ± 3
	TA97	TA98	TA100	TA102	TA1535
	With S9 metabolic activation				
Negative ^1^	13 ± 2	18 ± 1	97 ± 13	160 ± 18	16 ± 3
Positive ^2^	512 ± 30 *	174 ± 13 *	835 ± 50 *	718 ± 19 *	127 ± 18 *
PS23 (mg/plate)					
5	21 ± 2	14 ± 1	85 ± 18	148 ± 5	11 ± 3
2.5	17 ± 4	20 ± 3	114 ± 16	161 ± 17	12 ± 1
1.25	16 ± 3	20 ± 6	87 ± 13	164 ± 8	10 ± 2
0.625	16 ± 4	14 ± 4	79 ± 9	175 ± 8	13 ± 5
0.3125	21 ± 6	22 ± 4	106 ± 25	163 ± 11	12 ± 3

Data were expressed as mean ± SD (n = 3). The differences between the means were tested for statistical significance using one-way ANOVA followed by a Dunnett’s multiple comparison test. Differences between the negative control group and the other groups were considered statistically significant at *p* < 0.05 (*). ^1^ Solvent (distilled water) was used as negative control. ^2^ Positive control: w/ S9: all genotype used 2-aminoanthracene (10 μg/plate); w/o S9: 9-aminoacridine (105 μg/plate) for TA97; 4-nitroquinoline-N-Oxide (1 μg/plate) for TA98, TA100, and TA102; sodium azide (5 μg/plate) for TA1535.

**Table 3:** Chromosome aberration assay of PS23 in Chinese hamster ovary cells.

	Aberrant Cell (%) ^4^		Number of Cells with Structural Aberrations (%) ^3^						
	With Gap	Without Gap	G	B	D	R	g	b	e
3 h without S9 metabolic activation									
Negative ^1^	0.2 5 ± 0.50	0.25 ± 0.50	0.0 ± 0.0	0.0 ± 0.0	0.0 ± 0.0	0.0 ± 0.0	0.0 ± 0.0	0.25 ± 0.50	0.0 ± 0.0
Positive ^2^	8.25 ± 3.30 ***	6.25 ± 2.75 **	0.75 ± 0.50	2.00 ± 1.15 **	1.50 ± 1.29 *	0.0 ± 0.0	1.25 ± 0.50 *	2.00 ± 1.41	0.75 ± 0.96
PS23 (mg/mL)									
0.16	0.75 ± 1.50	0.50 ± 1.00	0.0 ± 0.0	0.25 ± 0.50	0.0 ± 0.0	0.0 ± 0.0	0.25 ± 0.50	0.25 ± 0.50	0.0 ± 0.0
0.313	1.00 ± 0.82	0.50 ± 0.58	0.25 ± 0.50	0.25 ± 0.50	0.0 ± 0.0	0.0 ± 0.0	0.25 ± 0.50	0.25 ± 0.50	0.0 ± 0.0
0.625	1.00 ± 1.15	0.75 ± 0.96	0.0 ± 0.0	0.0 ± 0.0	0.0 ± 0.0	0.0 ± 0.0	0.25 ± 0.50	0.75 ± 0.96	0.0 ± 0.0
3 h with S9 metabolic activation									
Negative ^1^	0.25 ± 0.50	0.0 ± 0.0	0.0 ± 0.0	0.0 ± 0.0	0.0 ± 0.0	0.0 ± 0.0	0.25 ± 0.50	0.0 ± 0.0	0.0 ± 0.0
Positive ^2^	11.00 ± 1.83 ***	8.00 ± 1.83 ***	1.00 ± 0.82	2.75 ± 0.50 ***	1.50 ± 0.58 ***	0.75 ± 0.96	2.00 ± 0.82 **	2.75 ± 0.96 ***	0.25 ± 0.50
PS23 (mg/mL)									
0.16	0.25 ± 0.50	0.25 ± 0.50	0.0 ± 0.0	0.0 ± 0.0	0.0 ± 0.0	0.0 ± 0.0	0.0 ± 0.0	0.25 ± 0.50	0.0 ± 0.0
0.313	0.25 ± 0.50	0.0 ± 0.0	0.0 ± 0.0	0.0 ± 0.0	0.0 ± 0.0	0.0 ± 0.0	0.25 ± 0.50	0.0 ± 0.0	0.0 ± 0.0
0.625	0.50 ± 0.58	0.0 ± 0.0	0.25 ± 0.50	0.0 ± 0.0	0.0 ± 0.0	0.0 ± 0.0	0.25 ± 0.50	0.0 ± 0.0	0.0 ± 0.0
24 h without S9 metabolic activation									
Negative ^1^	0.25 ± 0.50	0.25 ± 0.50	0.0 ± 0.0	0.0 ± 0.0	0.0 ± 0.0	0.0 ± 0.0	0.0 ± 0.0	0.25 ± 0.50	0.0 ± 0.0
Positive ^2^	9.75 ± 1.50 ***	8.25 ± 1.71 ***	1.00 ± 0.82	2.75 ± 0.50 ***	2.50 ± 1.29 **	0.25 ± 0.50	0.50 ± 0.58	3.00 ± 0.82 ***	0.50 ± 0.58
PS23 (mg/mL)									
0.16	1.00 ± 0.82	0.50 ± 0.58	0.25 ± 0.50	0.0 ± 0.0	0.0 ± 0.0	0.0 ± 0.0	0.50 ± 0.58	0.25 ± 0.50	0.0 ± 0.0
0.313	0.75 ± 0.96	0.0 ± 0.0	0.0 ± 0.0	0.25 ± 0.50	0.0 ± 0.0	0.0 ± 0.0	0.25 ± 0.50	0.0 ± 0.0	0.0 ± 0.0
0.625	0.3 ± 0.5	0.0 ± 0.0	0.25 ± 0.50	0.0 ± 0.0	0.0 ± 0.0	0.0 ± 0.0	0.50 ± 0.58	0.0 ± 0.0	0.0 ± 0.0

No significant aberration was shown under the treatment groups. Data are expressed as the mean ± SD. (n = 3). Significant difference as *: *p* < 0.05, ** *p* < 0.01, *** *p* < 0.001 was compared between the control and the treatment group by one-way ANOVA. ^1^ Solvent (distilled water) was used as the negative control. ^2^ Mitomycin C 1 µg/mL was used as the positive control in the condition without S9 metabolic activation; benzo(a)pyrene 5 g/mL was used as positive control in the condition with S9 metabolic activation. ^3^ G: chromosome gap; B: chromosome break; D: dicentric; R: ring; g: chromatid gap; b: chromatid break; e: exchange. ^4^ The chromatid and chromosome gaps were recorded but separated into two groups, with gaps and without gaps.

**Table 4:** Micronucleus test of PS23 on male ICR mice.

	Negative	PS23 (g/kg b.w.)			Positive
	Distilled Water	0.5	1.0	2.0	Cyclophosphamide 100 mg/kg
MNPCEs (‰)					
Day 1	0.6 ± 0.5	0.8 ± 0.8	0.2 ± 0.4	0.4 ± 0.5	3.2 ± 0.8 ***
Day 2	0.4 ± 0.5	0.6 ± 0.9	0.6 ± 0.5	0.6 ± 0.5	3.2 ± 0.8 ***
Day 3	0.6 ± 0.5	0.4 ± 0.5	0.4 ± 0.5	0.6 ± 0.9	2.8 ± 0.8 ***
PCEs (%)					
Day 1	6.4 ± 1.3	5.8 ± 1.3	5.4 ± 1.5	6.0 ± 1.4	5.2 ± 0.8
Day 2	5.4 ± 1.1	5.6 ± 1.8	5.4 ± 1.7	5.2 ± 1.6	5.4 ± 1.1
Day 3	6.6 ± 1.1	6.4 ± 1.1	7.2 ± 0.8	6.8 ± 1.3	5.6 ± 0.9

No significant change of micronuclei or reticulocytes was shown under the PS23 treatment groups. Data are expressed as the mean ± SD. (n = 5). Significant difference as *** *p* < 0.001 was compared between the control and the treatment group using one-way ANOVA. PCEs (%), ratio of polychromatic erythrocytes to total erythrocytes; MNPCEs (‰), frequency of micronucleated polychromatic erythrocytes.

**Table 5:** Hematology value changes in male and female ICR mice after oral administration of *L. paracasei* PS23 powder.

		Male				Female			
		0	40	400	4000	0	40	400	4000
Red blood cell count (RBC)	10^6^/dL	6.26 ± 3.81	5.12 ± 4.45	7.62 ± 3.42	7.33 ± 3.1	6.85 ± 3.75	8.23 ± 2.23	9.49 ± 0.54	7.21 ± 2.41
Hematocrit (HCT)	%	46.65 ± 2.45	46.25 ± 2.37	44.98 ± 8.34	43.56 ± 5.09	34.21 ± 18.17	42.23 ± 10.82	48.06 ± 2.36	36.18 ± 11.44
Hemoglobin (Hb)	g/L	146.5 ± 9	149 ± 9.47	142.13 ± 25.78	141.71 ± 11.05	126.8 ± 47.08	135 ± 44.98	154.5 ± 8.35	149.6 ± 13.08
Mean corpuscular hemoglobin (MCH)	Pg	15.2 ± 1.21	16.38 ± 1.42	15.39 ± 1.59	15.54 ± 0.33	27.94 ± 20.84	15.34 ± 4.36	16.29 ± 0.45	23.98 ± 10.53
MCH Concentration (MCHC)	g/dL	288.8 ± 48.66	319.4 ± 27.69	307.2 ± 32.29	330.9 ± 38.65	535.5 ± 374.67	299 ± 87.69	321.5 ± 11.06	469.5 ± 194.13
Mean corpuscular volume (MCV)	fL	52.31 ± 3.19	52.62 ± 3.79	50.16 ± 2.06	50.35 ± 1.93	51.02 ± 2.54	51.94 ± 2.79	50.69 ± 1.43	50.64 ± 1.76
RBC Distribution Width coefficient of variation (RDW-CV)	%	18.09 ± 3.21	17.06 ± 2.95	19.5 ± 2.58	18.92 ± 3.33	18.13 ± 2.85	19.1 ± 1.43	19.33 ± 0.56	17.32 ± 2.36
RBC Distribution Width standard deviation (RDW-SD)	fL	30.53 ± 3.23	28.78 ± 2.75	31.36 ± 2.29	30.77 ± 3.64	29.57 ± 2.2	31.46 ± 1.84	30.03 ± 1.3	28.42 ± 1.82
Platelet distribution width (PDW)	fL	8 ± 0.77	6.61 ± 0.72 *	7.96 ± 0.78	7.78 ± 0.83	7.82 ± 1.07	7.31 ± 0.64	7.48 ± 0.27	7.74 ± 0.39
Mean platelet volume (MPV)	fL	7.68 ± 0.61	6.76 ± 0.39 *	7.47 ± 0.56	7.42 ± 0.39	7.55 ± 0.3	7.08 ± 0.26 *	7.05 ± 0.25 *	7.48 ± 0.38
White blood cell count (WBC)	10^3^/L	6.16 ± 3.65	6.7 ± 2.49	7.26 ± 3.72	7.1 ± 3.04	5.57 ± 3.67	4.69 ± 1.51	7.92 ± 3.53	6.43 ± 4.46
Lymphocytes	%	76.9 ± 6.79	85.36 ± 3.24	84.61 ± 4.15	83.3 ± 4.3	75.65 ± 3.05	84.07 ± 5.77	82.42 ± 3.07	84.88 ± 2.64
Neutrophils	%	18.97 ± 5.77	11.1 ± 1.68	12.16 ± 3.67	14.3 ± 3.33	20.1 ± 3.2	11.7 ± 4.74	12.5 ± 3.59	10.63 ± 1.81
Monocytes	%	0.59 ± 0.54	0.68 ± 0.28	1.03 ± 0.74	0.73 ± 0.21	1.15 ± 0.15	0.33 ± 0.09	0.8 ± 0.38	0.58 ± 0.44
Eosinophil	%	2.77 ± 1.21	2.14 ± 1.96	1.69 ± 1.17	1.43 ± 0.65	1.85 ± 0.55	3.43 ± 1.14	3.74 ± 1.02	2.75 ± 1.77
Basophils	%	0.77 ± 0.38	0.46 ± 0.42	0.47 ± 0.54	0.47 ± 0.4	0.52 ± 0.45	1.05 ± 0.96	0.48 ± 0.48	1.09 ± 0.93
Platelets count (PLT)	10^6^/L	663.6 ± 511.66	545.8 ± 549.61	1188.5 ± 782.7	803.8 ± 464.88	490.5 ± 372.8	538 ± 188.46	743.3 ± 168.38	455.9 ± 212.25
Platelet Large Cell Ratio (P-LCR)	%	9.75 ± 4.09	6.75 ± 3.41	8.75 ± 2.68	7.72 ± 2.41	8.88 ± 2.7	6.45 ± 1.64 *	6.03 ± 1.24 *	8.2 ± 2.24
Plateletcrit (PCT)	%	0.5 ± 0.37	0.36 ± 0.36	0.88 ± 0.59	0.6 ± 0.34	0.37 ± 0.28	0.38 ± 0.14	0.52 ± 0.12	0.34 ± 0.15

Data were expressed as mean ± S.D. Significant difference between the control and treatment group at *: *p* < 0.05 was compared by one-way ANOVA.

**Table 6:** Biochemical analysis of subacute toxicity after 28-day PS23 treatment on ICR mice.

		Male				Female			
DAILY DOSE (mg/kg(b.w/day))		0	40	400	4000	0	40	400	4000
Calcium	mg/dL	7.24 ± 0.81	7.11 ± 0.74	7.63 ± 1.75	8.14 ±1.25	8.36 ± 0.64	9.22 ± 1.25	9.39 ± 1.64	9.04 ± 1.08
Chloride	mmol/L	89.00 ± 2.16	84.90 ± 2.60 *	85.80 ± 2.35 *	86.20 ± 1.40 *	79.82 ± 24.95	84.30 ± 3.37	86.40 ± 0.97	87.20 ± 1.55 *
Phosphorus	mg/dL	7.48 ± 0.91	7.24 ± 1.08	7.64 ± 0.72	8.93 ± 1.54 *	5.90 ± 1.07	6.93 ± 0.89	7.06 ± 0.93	7.34 ± 0.63
Potassium	mmol/L	3.68 ± 0.40	3.73 ± 0.33	3.34 ± 0.33	3.31 ± 0.39	4.24 ± 0.42	4.36 ± 0.34	4.07 ± 0.33	3.81 ± 0.30
Sodium	mmol/L	134.60 ± 2.76	129.60 ± 3.44 *	133.00 ± 2.62	133.30 ± 1.77	133.70 ± 1.77	129.80 ± 5.29 *	133.20 ± 2.44	133.00 ± 2.26
Glucose	mg/dL	120.00 ± 18.62	95.80 ± 20.00 *	84.80 ± 18.24 *	119.70 ± 27.33	106.10 ± 17.44	109.40 ± 23.28	109.90 ± 28.89	122.3 ± 37.5
Total Bilirubin (TBIL)	mg/dL	0.16 ± 0.05	0.14 ± 0.07	0.18 ± 0.09	0.13 ± 0.05	0.39 ± 0.12	0.36 ± 0.07	0.39 ± 0.10	0.39 ± 0.07
Alanine aminotransferase (ALT)	U/L	32.20 ± 3.94	34.00 ± 34.88	28.00 ± 18.59	20.10 ± 4.04	19.00 ± 6.13	17.60 ± 5.46	18.50 ± 7.71	15.00 ± 4.24
Aspartate aminotransferase (AST)	U/L	85.2 ± 23.40	56.80 ± 26.88	73.40 ± 47.41	46.30 ± 13.62 *	71.80 ± 28.23	60.90 ± 16.41	69.60 ± 32.89	59.60 ± 10.62
Alkaline phosphatase (ALP)	U/L	48.10 ± 10.03	52.2 ± 9.93	45.8 ± 10.16	51.4 ± 13.53	193.40 ± 76.69	254.30 ± 52.81	216.10 ± 60.00	164.80 ±48.50 *
Creatinine	mg/dL	0.24 ± 0.05	0.21 ± 0.03	0.21 ± 0.03	0.21 ± 0.03	0.20 ± 0.16	0.15 ± 0.05	0.16 ± 0.05	0.16 ± 0.05
Blood urea nitrogen (BUN)	mg/dL	31.61 ± 3.65	31.66 ± 2.82	31.88 ± 3.41	30.60 ± 2.79	25.75 ± 4.99	23.02 ± 3.44	23.42 ± 3.96	22.76 ± 4.42
Albumin	g/dL	2.09 ± 0.23	1.90 ± 0.11	1.93 ± 0.22	2.09 ± 0.32	2.13 ± 0.32	2.35 ± 0.25	2.31 ±0.55	2.15 ± 0.19
Total protein	g/dL	4.58 ± 0.32	4.34 ± 0.25	4.53 ± 0.33	4.69 ± 0.53	4.39 ± 0.35	4.67 ± 0.41	4.69 ± 0.83	4.38 ± 0.39
Cholesterol	mg/dL	152.20 ± 23.86	116.90 ± 18.33 *	128.10 ± 32.91	149.20 ± 38.44	85.40 ± 23.55	103.60 ± 17.51	82.80 ± 27.53	92.80 ± 18.47
Triglycerides	mg/dL	119.50 ± 38.02	92.70 ± 28.28	134.10 ± 44.30	137.70 ± 38.26	141.90 ± 61.07	146.90 ± 25.27	131.20 ± 37.45	134.10 ± 44.57
Lactate dehydrogenase (LDH)	U/L	677.90 ± 186.67	829.50 ± 98.58	771.00 ± 155.25	558.20 ± 277.16	736.00 ± 137.37	623.60 ± 192.60	680.10 ± 204.08	526.40 ± 154.84
Amylase	U/L	1028.20 ± 174.35	698.60 ± 79.99 *	880.30 ± 201.17	942.50 ± 110.29	940.20 ± 216.34	795.70 ± 156.60	784.80 ± 184.10	681.00 ± 110.49

Data were expressed as mean ± S.D. Significant difference between the control and treatment group at *: *p* < 0.05 was compared by one-way ANOVA.

**Table 7:** Organ weight changes in male and female ICR mice after oral administration of *L. paracasei* PS23 powder.

		Male				Female			
DAILY DOSE (mg/kg)		0	40	400	4000	0	40	400	4000
ADRENALS									
Absolute weight	mg	8.92 ± 3.28	6.32 ± 2.69	7.48 ± 2.87	7.18 ± 2.22	9.87 ± 1.08	10.64 ± 2.82	9.99 ± 1.52	9.98 ± 3.07
Ratio per body weight	(10^−3^)	0.02 ± 0.01	0.02 ± 0.01	0.02 ± 0.01	0.02 ± 0.01	0.03 ± 0	0.04 ± 0.01	0.04 ± 0.01	0.03 ± 0.01
HEART									
Absolute weight	mg	181 ± 35.1	160.5 ± 12.57	164.5 ± 25.65	174 ± 22.71	126.99 ± 14.74	116.45 ± 10.29	116.97 ± 10.81	126.1 ± 11.78
Ratio per body weight	(10^−3^)	0.49 ± 0.09	0.43 ± 0.03	0.44 ± 0.08	0.45 ± 0.06	0.44 ± 0.04	0.41 ± 0.03	0.41 ± 0.04	0.41 ± 0.04
KIDNEYS									
Absolute weight	mg	578 ± 92.59	539 ± 51.09	485.5 ± 42.72	553.5 ± 59.07	358.53 ± 49.41	328.8 ± 22.28	329.02 ± 26.37	343.48 ± 40.6
Ratio per body weight	(10^−3^)	1.55 ± 0.21	1.46 ± 0.1	1.3 ± 0.14	1.43 ± 0.2	1.23 ± 0.15	1.16 ± 0.05	1.16 ± 0.09	1.12 ± 0.12
LIVER									
Absolute weight	mg	1745 ± 265.55	1523 ± 119.63 *	1510 ± 116.43 *	1759 ± 179.72	1348.59 ± 177.39	1144.98 ± 122.42 *	1187.37 ± 127.53	1253.72 ± 191.86
Ratio per body weight	(10^−3^)	4.72 ± 0.82	4.12 ± 0.34 *	4.04 ± 0.27 *	4.5 ± 0.33	4.64 ± 0.59	4.04 ± 0.37 *	4.19 ± 0.39	4.07 ± 0.5 *
SPLEEN									
Absolute weight	mg	92 ± 11.35	76.7 ± 15.71	96.4 ± 18.41	101.82 ± 11.86	96.88 ± 11.13	85.59 ± 11.28	91.73 ± 14.24	110.02 ± 29.12
Ratio per body weight	(10^−3^)	0.25 ± 0.03	0.21 ± 0.04	0.26 ± 0.04	0.26 ± 0.03	0.33 ± 0.04	0.3 ± 0.04	0.32 ± 0.05	0.36 ± 0.08
TESTIS/OVARY									
Absolute weight	mg	231 ± 30.35	233.87 ± 39.81	232 ± 24.4	219 ± 37.55	21.63 ± 5.94	16.86 ± 4.6	21.01 ± 3.79	23.81 ± 4.46
Ratio per body weight	(10^−3^)	0.62 ± 0.1	0.63 ± 0.09	0.62 ± 0.07	0.56 ± 0.11	0.08 ± 0.02	0.06 ± 0.02	0.07 ± 0.01	0.08 ± 0.01
Epididymis/Uterus									
Absolute weight	mg	37.34 ± 7.46	33.08 ± 6.97	32.32 ± 5.35	33.12 ± 9.94	122.82 ± 35.68	82.93 ± 30.75	124.72 ± 47.92	141.4 ± 52.16
Ratio per body weight	(10^−3^)	0.1 ± 0.02	0.09 ± 0.02	0.09 ± 0.01	0.08 ± 0.03	0.42 ± 0.13	0.29 ± 0.11	0.44 ± 0.18	0.46 ± 0.16
LUNG									
Absolute weight	mg	215 ± 29.15	194 ± 11.74	194 ± 23.19	211 ± 16.63	176.03 ± 18.38	165.95 ± 11.1	162.37 ± 12.4	179.98 ± 14.83
Ratio per body weight	(10^−3^)	0.58 ± 0.07	0.53 ± 0.04	0.52 ± 0.08	0.54 ± 0.06	0.61 ± 0.06	0.59 ± 0.03	0.58 ± 0.07	0.59 ± 0.05

Data were expressed as mean ± S.D. Significant difference between the control and treatment group at *: *p* < 0.05 was compared by one-way ANOVA.

## Data Availability

Not applicable.

## Supplementary Materials

The supporting information can be downloaded at: https://www.mdpi.com/article/10.3390/ijms24010724/s1.
