# Supplementary material for: Safety Evaluation and Anti-Inflammatory Efficacy of Lacticaseibacillus paracasei PS23
Source: Int J Mol Sci. 2022 Dec 31;24(1):724. doi: 10.3390/ijms24010724 (PMC9821173; doi:10.3390/ijms24010724)
Supplement: Supplementary file 1 [file ijms-24-00724-s001.zip › ijms-2069243-supplementary.pdf]

Supplementary File

MATERIALS and METHODS:

1. **Genome sequencing and annotation:**

   Whole genome sequencing of *Lactobacillus paracasei* subsp. *paracasei* PS23 was performed using the Pacific Biosciences RSII instrument in accordance with the manufacturer's instructions. To construct the PacBio library and for subsequent sequencing, genomic DNA was sheared using a Covaris g-TUBE followed by purification via binding to pre-washed AMPure PB beads (Part Number: PB100-265-900). Following end-repair, the blunt adapters underwent ligation followed by exonuclease incubation for the removal of all un-ligated adapters and DNA. The final "SMRT bells" were annealed using primers and bound to a proprietary polymerase using the PacBio DNA/Polymerase Binding Kit P6 v2 (Part Number PB100-372-700) to form a "binding complex". Following dilution, the PacBio library was loaded onto the instrument using the DNA Sequencing Kit 4.0 v2 (Part Number PB100-612-400) and a SMRT Cell 8Pac for sequencing. Primary filtering analysis was performed using the RS instrument and secondary analysis was performed using the SMRT analysis pipeline, version 2.3.0. The longest reads were used as alignment seeds in the multi-molecule consensus error correction step of the hierarchical genome assembly process (HGAP) (Chin et al. 2013). The genome annotation was performed by using the NCBI Prokaryotic Genome Annotation Pipeline (PGAP-5.0, 2021-01-11.build5132) (Tatusova et al. 2016).

2. **Analysis of virulence factors:**

   Whole genome sequence of the strain was compared against up-to-date virulence factors databases for the existence of known pathogenic genes and toxin-producing gene using BLASTp or BLASTn. The cut-off values were as follows: e-value <1.0e-5, >60% coverage, >70% identity. The databases used for comparison were MvirDB (Zhou et al. 2007) (last updated: 2012.04.22), VFDB (last updated: 2021.03.26) (Chen et al. 2016), CGE VirulenceFinder 2.0 (database 2020-05-29) (Joensen et al. 2014), CGE PathogenFinder 1.1 (2014-03-19) (Cosentino et al. 2014) and PAIDB 2.0 (Yoon et al. 2015). All hits were reviewed to predict possible functions and the closest organism for concluding the pathogenicity risk of gene.

3. **Analysis of antibiotic resistance genes:**

The resistance to antibiotics was analyzed using the whole genome sequence of the strain by following programs with the default criteria. The programs and databases used for analysis were NCBI AMRFinderPlus (AMRFinderPlus 3.10.1, database 2021-03-01.1) (Feldgarden et al. 2019), CARD (RGI 5.1.1, CARD 3.1.1) (Alcock et al. 2020), ResFinder (ResFinder 4.1, database 2021-02-19) (Zankari et al. 2012) and ARG-ANNOT (BLASTp and BLASTn, e-value <1.0e-5, >60% coverage, >70% identity) (V6 July 2019) (Gupta et al. 2014).

4. Detection of biogenic amine producing genes:

Microbial biogenic amine producing genes of histidine decarboxylase, tyrosine decarboxylase, ornithine decarboxylase, agmatine deiminase, lysine decarboxylase were collected from NCBI GenBank, and the BLAST databases were constructed. Whole genome sequence of the strains was compared against the blast databases for existences of those known genes responsible for the production of biogenic amines by BLASTp and BLASTn. The cut-off values were as follows: e-value (1.0e-5), coverage (60%), and identity (70%). In addition, the BlastKOALA search tool in the Kyoto Encyclopedia of Genes and Genomes (KEGG) database (Release 98.0) was used to confirm biogenic amine producing genes.

RESULTS and DISCUSSION:

1. Sequencing statistics

Sequencing statistics including total raw base and the number of reads for strain PS23 obtained via PacBio was summarized in Table S1.

2. General features of de novo assembly

The assembled genome consisted of 2 contigs (1 chromosome, 1 plasmid-like contig) with a genome size of 3.01 Mb, GC content of 46.3%. Gene prediction and annotation results indicated that the genome contains 2,890 protein coding genes, 15 rRNAs and 59 tRNAs (Table S2). A detailed description of gene annotation was presented in Annex.

3. Antimicrobial resistance genes analysis

Whole genome of strain PS23 was compared with NCBI AMRFinderPlus (AMRFinderPlus 3.10.1, database 2021-03-01.1), CARD (RGI 5.1.1, CARD 3.1.1), ResFinder (ResFinder 4.1, database 2021-02-19) and ARG-ANNOT (V6 July 2019). The results showed that all predicted genes were not homologous to known

antimicrobial resistance genes (Table S3).

4. **Virulence factors analysis**

Pathogenicity analyses for strain PS23 were performed by comparing against up-to-date databases (MvirDB last updated: 2012.4.22, VFDB last updated: 2021.03.26, CGE VirulenceFinder 2.0 database 2020-05-29, CGE PathogenFinder 1.1 2014-03-19 and PAIDB 2.0). The results concluded that all predicted genes showed no homologous to known virulence factors (Table S4).

5. **Biogenic amine production genes analysis**

Whole genome sequence of strain PS23 was compared against the blast databases for any existences of known biogenic amine producing genes. The cut-off values were as follows: e-value (1.0e-5), coverage (60%), and identity (70%). In addition, the BlastKOALA search tool in the Kyoto Encyclopedia of Genes and Genomes (KEGG) database (Release 98.0) was used to confirm biogenic amine producing genes. The results showed that one gene was homologous to known ornithine decarboxylase, which might involve in the biosynthesis of putrescine (Table S5).

REFERENCES:

1. Alcock B. P., Raphenya A. R., Lau T. T. Y., et al. 2020. CARD 2020: antibiotic resistome surveillance with the comprehensive antibiotic resistance database. Nucleic Acids Res. 48:D517-D525.
2. Benjamin B , Chao X, Daniel HH. 2015. Fast and Sensitive Protein Alignment using DIAMOND. Nat Methods. 12: 59- 60.
3. Chen LH, Zheng DD, Liu B, Yang J and Jin Q. 2016. VFDB 2016: hierarchical and refined dataset for big data analysis-10 years on. Nucleic Acids Res. 44:D694-D697.
4. Chin, C. S., Alexander, D. H., Marks, P., Klammer, A. A., Drake, J., Heiner, C., Clum, A., Copeland, A., Huddleston, J., Eichler, E. E., Turner, S. W. and Korlach, J. 2013. Nonhybrid, finished microbial genome assemblies from long-read SMRT sequencing data. Nat. Methods 10 563–569.
5. Cosentino, S., Voldby Larsen M, Møller Aarestrup F, Lund O 2013. PathogenFinder - Distinguishing Friend from foe Using Bacterial Whole Genome Sequence Data. PLoS ONE 8(10): e77302.
6. Delcher AL, Harmon D, Kasif S, White O, Salzberg SL. 1999. Improved microbial gene identification with GLIMMER. Nucleic Acids Res. 27:4636-4641.
7. Feldgarden, M., Brover, V., Haft, D.H., Prasad, A.B., Slotta, D.J., Tolstoy, I., Tyson, G.H., Zhao, S., Hsu, C.-H., McDermott, P.F., Tadesse, D.A., Morales, C., Simmons, M., Tillman, G., Wasilenko, J., Folster, J.P., Klimke, W., 2019. Validating the NCBI AMRFinder Tool and Resistance Gene Database Using Antimicrobial Resistance Genotype-Phenotype Correlations in a Collection of NARMS Isolates. Antimicrob. Agents Chemother. pii: AAC.00483-19.
8. Gupta SK, Padmanabhan BR, Diene SM, Lopez-Rojas R, Kempf M, Landraud L, Rolain JM. 2014. ARG-ANNOT, a new bioinformatic tool to discover antibiotic resistance genes in bacterial genomes. Antimicrob Agents Chemother. 58:212-20.
9. Joensen KG, Scheutz F, Lund O, Hasman H, Kaas RS, Nielsen EM, Aarestrup FM. 2014. Real-time whole-genome sequencing for routine typing, surveillance, and outbreak detection of verotoxigenic Escherichia coli. J. Clin. Micobiol. 52: 1501-1510.
10. Tatusova T, DiCuccio M, Badretdin A, Chetvernin V, Nawrocki EP, Zaslavsky L, Lomsadze A, Pruitt KD, Borodovsky M, Ostell J. 2016. NCBI Prokaryotic Genome Annotation Pipeline. Nucleic Acids Res 44:6614–6624.
11. Yoon SH, Park YK, and Kim JF. 2015. PAIDB v2.0: exploration and analysis of pathogenicity and resistance islands Nucleic Acids Res. 43:D624-D630.
12. Zankari E, Hasman H, Cosentino S, Vestergaard M, Rasmussen S, Lund O, Aarestrup FM, Larsen MV. 2012. Identification of acquired antimicrobial resistance genes. J Antimicrob Chemother. 67: 2640-2644.
13. Zhou CE, Smith J, Lam M, Zemla A, Dyer M D, Slezak T. 2007. MvirDB--a microbial database of

protein toxins, virulence factors and antibiotic resistance genes for bio-defence applications. Nucleic Acids Res. 35:D391-394.

FIGURES and TABLES

Table S1. PacBio sequencing statistics of strain PS23.

Sequencing statistics	
Number of raw reads	60,816
Total raw bases (Mb)	498.8
Average read length	8202
Quality	0.82

Table S2. General genome features of strain PS23.

Genome statistics	
Number of contigs	2 (including 1 chromosome and 1 plasmid-like contig)
Genome length (bp)	3,014,784
N50 (bp)	2,939,246
Coverage	400
GC (%)	46.3 (unitig_0: 46.4, unitig_1: 43.9)
Genes	2,890
CDSs	2,813
rRNA	15
tRNAs	59

Table S3. Antimicrobial resistance genes analyses of strain PS23.

Database	Gene_id	Location	Hit	Known species	E-value	Identity (%)	Coverage (%)	Possible function
CARD	n.d.*							
AMRFinderPlus	n.d.*							
ResFinder	n.d.*							
ARG-ANNOT	n.d.*							

* Antimicrobial resistance gene was not detected.

Table S4. Virulence factors analyses of strain PS23.

Database	Gene_id	Location	Hit	Known species	E-value	Identity (%)	Coverage (%)	Possible function
VFDB	n.d.*							
CGE VirulenceFinder	n.d.*							
CGE PathogenFinder	n.d.*							
MvirDB	n.d.*							
PAI DB	n.d.*							

* Virulence factor was not detected.

Table S5. Biogenic amine production genes analyses of strain PS23.

Target	Gene_id	Hit	Known species	E-value	Identity (%)	Coverage (%)
Histidine decarboxylase [EC:4.1.1.22]	n.d.*					
Tyrosine decarboxylase [EC:4.1.1.25]	n.d.*					
Ornithine decarboxylase [EC:4.1.1.17]	PS23_002482	WP_165846787.1	*Lacticaseibacillus paracasei*	0.0	99.57	100
Agmatine deiminase [EC:3.5.3.11]	n.d.*					
Lysine decarboxylase [EC:4.1.1.18]	n.d.*					

* Biogenic amine production gene was not detected.

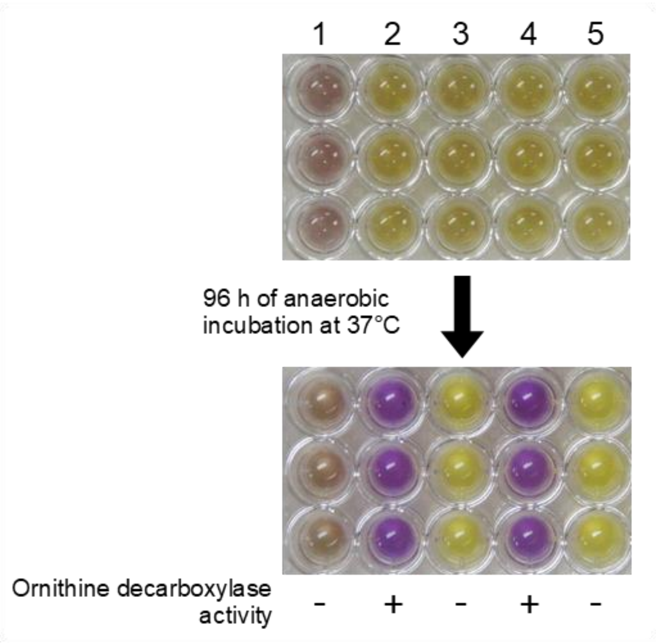

Figure S1. Ornithine decarboxylase activity of lactobacilli. Overnight grown cultures of lactobacilli in MRS broth 1% (v/v) inoculated into wells containing Moeller decarboxylase broth supplemented with 2% L-ornithine (pH 6.0 ± 0.2). The wells were overlayed with sterile mineral oil, and then the 96-well plate was incubated anaerobically at 37 °C for 96 h as described in the methods. Formation of putrescine from ornithine increases the pH of the medium, changing the color of the indicator to purple. This result is representative of three independent experiments performed in triplicate. Lanes: 1, 1% fresh MRS broth as a blank control. 2, *L. saerimneri* 30a (ATCC 33222) as a positive control. 3, *L. casei* ATCC 393^{T}; 4, *L. paracasei* ATCC 25302^{T}; 5, *L. paracasei* PS23. Ornithine decarboxylase activity is indicated at the bottom.
